# Effects of dietary tryptophan supplementation on rectal temperature, humoral immunity, and cecal microflora composition of heat-stressed broilers

**DOI:** 10.3389/fvets.2023.1247260

**Published:** 2023-09-28

**Authors:** Qiufen Li, Jingxin Ouyang, Chenxi Deng, Hua Zhou, Jinming You, Guanhong Li

**Affiliations:** ^1^Jiangxi Province Key Laboratory of Animal Nutrition, College of Animal Science and Technology, Jiangxi Agricultural University, Nanchang, China; ^2^Jiangxi Province Key Innovation Center of Integration in Production and Education for High-quality and Safe Livestock and Poultry, Nanchang, China; ^3^Institute of Veterinary Drug, Jiangxi Agricultural University, Nanchang, China

**Keywords:** tryptophan, heat stress, broilers, immunity, cecal microflora

## Abstract

This trial aimed to determine the effects of tryptophan (Trp) on the rectal temperature, hormone, humoral immunity, and cecal microflora composition in broiler chickens under heat stress (HS). One hundred and eighty 18 days-old female Arbor Acres broilers were randomly divided into three treatment groups, with six replicates of ten birds in each replicate. The broilers were either raised under thermoneutral conditions (TN, 23 ± 1°C) or subjected to heat stress (34 ± 1°C for 8 h daily). The TN group received a basal diet, and another two heat-stressed groups were fed the basal diet (HS) or the basal diet supplemented with 0.18% Trp (HS + 0.18% Trp) for 21 consecutive days. The basal diet contained 0.18% Trp. Results revealed that HS increased the rectal temperature, serum epinephrine (EPI), and corticotropin-releasing hormone (CRH) concentrations (*p* < 0.05), reduced the bursal index, the levels of serum immunoglobulin A (IgA), IgG, IgM, and serotonin (5-HT) as well as the relative abundance of Actinobacteria in cecum (*p* < 0.05) compared with the TN group. Dietary supplementation of Trp decreased the rectal temperature, serum dopamine (DA), EPI, and the levels of CRH and L-kynurenine (*p* < 0.05), increased the bursal index, the levels of serum IgA, IgM, and 5-HT as well as the relative abundance of *Ruminococcus torques group* in cecum of heat-stressed broilers (*p* < 0.05) compared to HS group. In conclusion, dietary Trp supplementation decreased rectal temperature, improved cecal microbiota community and Trp metabolism, and enhanced humoral immunity of heat-stressed broilers.

## 1. Introduction

The autonomic nervous system (ANS) and hypothalamic–pituitary–adrenocortical (HPA) axis get activated under heat stress, which leads to the secretion of corticosterone (CORT) and catecholamines, resulting in neuroendocrine disorders and dysfunctions (1, 2). Heat stress elevates body temperature, causes oxidative stress, and affects intestinal barrier capabilities and immunological functions, consequently causing a decline in the growth performance of poultry and leading to huge economic losses for the global poultry industry (3, 4). Therefore, it is necessary to develop measures to relieve the negative impact of HS. Recent research has revealed that the HS in broiler chickens could be effectively alleviated by nutritional regulation (5).

Tryptophan (Trp) is metabolized through three primary pathways, including the kynurenine (KYN), serotonin (5-HT), and microbial pathways (6). The KYN pathway is the primary means of Trp metabolism, and it involves two rate-limiting enzymes, namely liver-derived tryptophan 2,3-dioxygenase (TDO) and extrahepatic indoleamine 2,3-dioxygenase (IDO) (7, 8). This KYN pathway plays a significant role in immunological responses, neural functions, and gut homeostasis, while abnormal activation of the KYN pathway is a cause of histopathological damage as well as impaired immune function (8, 9). In this process, abnormal activation of IDOs and TDOs against infection, inflammatory stimuli, and other stressful conditions result in the depletion of Trp and the excessive accumulation of certain metabolites, particularly those with cytotoxic effects, in the KYN metabolic pathway (10, 11). Trp concentration in the brain is unsaturated under normal physiological conditions, while tryptophan hydroxylase (TPH) is a rate-limiting enzyme for the synthesis of 5-HT. Therefore, diets or plasma that increase the Trp or large neutral amino acid (LNAA) ratio might have the ability to increase Trp concentration in the brain and consequently increase levels of central 5-HT (6). Studies on animals have demonstrated that the addition of Trp to the diet increased the expression of TPH1 in broilers and mice with chronic unpredictable mild stress, elevated the level of 5-HT in peripheral blood, and reduced the stress response (12). 5-HT plays a crucial regulatory role in the animal HPA axis by regulating the release of hypothalamic adrenocorticotropin-releasing factor (13, 14), and regulating behavioral and neuroendocrine responses to stress (15, 16). Although the host metabolic pathways are the primary pathway for the metabolism of Trp, metabolites produced by the gut microbiota from Trp (indole pathway) play a significant role in regulating the intestinal barrier function, and abnormal metabolism pathway is an important contributor to intestinal homeostasis imbalance (8, 17).

Trp pathways are known to change as a result of stress, which in turn affects the ability of the body to respond to stress (18). The modifications of the three important metabolic pathways of Trp in heat-stressed poultry and the function and mechanism in histopathological injury have not been documented. Therefore, the purposes of this study were to investigate the Trp metabolism changes in broiler chickens under HS as well as the effects of dietary Trp supplementation on the rectal temperature, hormone levels, immune function, and gut microbial composition in broilers under HS.

## 2. Materials and methods

All experimental broilers used in this study were prepared according to experimental procedures by the Laboratory Animal Ethics Committee of Jiangxi Agricultural University (JXAULL-2021-036).

### 2.1. Animals and experimental design

One hundred and eighty 18 days-old female Arbor Acres broilers with similar weight (481.50 ± 26.68 g) were randomly divided into three treatment groups, with six replicates of ten birds in each replicate. The birds in the TN group were fed the basal diet and raised under the thermoneutral condition (23 ± 1°C); The birds in the HS group were fed the basal diet and raised under HS (HS, 34 ± 1°C for 8 h daily); The birds in the HS + 0.18% Trp group were fed the basal diet supplemented with 0.18%Trp and raised under HS. The experiment lasted 21 days with an adaption period of 3 days. Relative humidity was kept between 65 and 70%. The birds were allowed *ad libitum* access to water and feed throughout the experiment period. The basal diet (Table 1) was formulated according to the NRC (1994) nutrient recommendations. The Trp level in the basal diet was 0.18%. L-Glutamic acid was added as an isonitrogenous control (19–21).

**Table 1:** Ingredients and nutrient compositions of the experimental diets (as-fed basis).

Item	Day 1–18	Day 19–42		
		TN	HS	HS + 0.18%Trp
Ingredient (%)				
Corn	52.50	57.00	57.00	57.00
Soybean meal (CP, 43%)	23.00	16.20	16.20	16.20
Corn gluten meal	10.00	10.00	10.00	10.00
Extruded soybean (CP, 34%)	6.00	8.00	8.00	8.00
Soybean oil	2.50	3.50	3.50	3.50
NaCl	0.30	0.30	0.30	0.30
CaCO_3_	1.50	1.50	1.50	1.50
Calcium hydrogen phosphate	1.70	1.50	1.50	1.50
Mineral premix^a^	0.20	0.20	0.20	0.20
L-Lysine HCl	0.20	0.31	0.31	0.31
DL-Methionine	0.15	0.10	0.10	0.10
Vitamin premix^b^	0.03	0.03	0.03	0.03
L-Tryptophan	0.00	0.00	0.00	0.18
L-Glutamic acid	0.00	0.39	0.39	0.13
Choline chloride	0.10	0.10	0.10	0.10
Zeolite powder	1.82	0.87	0.87	0.95
Total	100.00	100.00	100.00	100.00
Calculated nutrient composition (%)				
Metabolizable energy (MJ/kg)	12.81	13.40	13.40	13.40
Crude protein	22.38	20.48	20.48	20.48
Tryptophan^c^	0.23	0.20(0.18)	0.20(0.18)	0.38(0.33)
Lysine	1.16	1.11	1.11	1.11
Methionine	0.56	0.48	0.48	0.48
Ca	1.05	0.99	0.99	0.99
Available P	0.46	0.41	0.41	0.41

a Mineral premix provided the following per kilogram of diet: Fe (FeSO_4_ · H_2_O), 80 mg; Se (Na_2_SeO_3_), 0.15 mg; Cu (Cu_2_(OH)_3_Cl), 8 mg; Mn (MnSO_4_·H_2_O), 60 mg; Zn (ZnSO_4_·H_2_O), 40 mg and I (Ca(IO_3_)_2_), 0.35 mg.

b Vitamin premix provided the following per kilogram of diet: Vitamin A, 12000 IU; Vitamin D_3_, 3,000 IU; Vitamin E, 36 IU; Vitamin K_3_, 0.9 mg; Vitamin B_1_, 0.6 mg; Vitamin B_2_, 2.4 mg; Vitamin B_6_, 1.8 mg; Vitamin B_12_; 15 μg; D-biotin, 0.18 mg; D-pantothenic acid, 3 mg; Niacinamide, 38 mg; Folic acid, 0.75 mg.

c Data in parentheses indicate the analyzed values.

### 2.2. Sample collection and preparation

On days 28, 35, and 41, the rectal temperature of broilers was determined using a mercury thermometer. At 42 days of age, broilers blood was drawn from the wing vein one bird from six replicates in three groups, and the serum was separated by centrifugation at 3500 rpm for 10 min at 4°C and stored at −20°C. Following the collection of blood samples, birds were euthanized by cervical dislocation. Furthermore, the liver, spleen, thymus, and bursa were collected and weighed, and the immune organ index was calculated. Samples of the hypothalamus, and liver were obtained and immediately stored in sterile tubes. The samples obtained were frozen in liquid nitrogen and maintained at −80°C until further analysis. The cecum contents for 16S rDNA sequencing were performed according to the manufacturer’s instructions (Shanghai Zhongke New Life Biotechnology Co., Ltd. Shanghai, China).

### 2.3. Determination of the serum immunoglobulin and hormone levels

The enzyme-linked immune sorbent assay (ELISA) (MLBIO Co., Ltd. Shanghai, China) was employed to determine the levels of serum IgA, IgG, IgM, CORT, dopamine (DA), epinephrine (EPI), noradrenaline (NOR), adrenocorticotropic hormone (ACTH), and corticotropin-releasing hormone (CRH).

### 2.4. Quantitative real-time PCR

Frozen liver and intestinal samples (100 mg) were subjected to RNA extraction using TransZol Up Plus RNA Kit (ER501, TransGen Biotech, Beijing, China). RNA quality and concentration were evaluated using the ultra-micro-nucleic acid protein tester (BioDrop, Cambridge, United Kingdom). Reverse-transcription was performed using EasyScript^®^ One-Step gDNA Removal and cDNA Synthesis SuperMix (AE311, TransGen Biotech, Beijing, China). Quantitative RT-PCR (qPCR) was performed using TransStart^®^ Tip Green qPCR SuperMix (AQ601, TransGen Biotech, China). β-actin was employed as a frame of reference internally. Relative expression levels were calculated using the ΔΔCT method (22). The primer sequence information for all genes is listed in Table 2.

**Table 2:** Primer sequences of the reference genes and target genes.

Gene	Primer sequence (5′–3′)	GenBank ID
β-actin	Forward: GATTTCGAGCAGGAGATGGC	L08165.1
	Reverse: GCCAATGGTGATGACCTGAC	
TDO	Forward: ACTCCAGGACTTGAGGCAGA	XM_420377.5
	Reverse: CTGATTCTGGTTTGGCCTGT	
IDO-L1	Forward: GAGAAGGAGGAGAGCCCACT	XM_424397.5
	Reverse: CCAAACGCTGTAGGAAGGTG	
IDO-2 L	Forward: TGCACAGAACCAACCCACT	XM_015302448.1
	Reverse: ATGTACTGGGGACAGCCAGA	
KYAT	Forward: TAAAGATGGCGGGTGCAAAG	XM_004936631.3
	Reverse: CTTGCCGATAGGGTTGTGTG	
KMO	Forward: CGTCAATTCGACGTGGTTCA	XM_004942566.3
	Reverse: GGTAGAAGGCATGAGGGTGT	
TPH1	Forward: TCTATGAACCCGACAGAGCA	NM_204956.1
	Reverse: TCAGAcCCGTACATCAGCAC	
TPH2	Forward: AACCCATTCCCAGAGTGGAG	NM_001001301.1
	Reverse: ATCTTCCAGCTGAGGCACAT	
AANAT	Forward: TTCGAGATCGAGAGGGAAGC	NM_205158.1
	Reverse: AGGAAGTGGCGGATCTCATC	
MAO	Forward: CATGCCAATCAGGCACAGAA	NM_001030799.1
	Reverse: CCACACAGTGTTGCTACTCG	
SERT	Forward: GTTCTACGGCATCACCCAGT	AY573844.1
	Reverse: GCAAGTGACAAACAGCAGGA	
ATP1A1	Forward: CAAGGGAGTTGGCATCATCT	NM_205521.1
	Reverse: GAGCCATGAACAACACATGC	
SLC3A1	Forward: AAGGAGCTGAAGGGCTTACC	XM_004935370.2
	Reverse: CTTGGCTGCTGGTGTCAGTA	
SLC6A14	Forward: CCTGCTCATCTTGTTGGTGA	XM_015278436.1
	Reverse: GCATCTTTCCAAACCTCTGC	
SLC6A19	Forward: GAGTTGAAGGAACCGGCTTA	XM_419056.5
	Reverse: ATGACAAACCCAGGCAGAAG	

### 2.5. Hypothalamus Trp, 5-HT, and 5-HIAA levels

The hypothalamus tissues were prepared as a 10% tissue homogenate in PBS and centrifuged at 5000 rpm for 10 min to collect the supernatant. The protein concentration was determined using the bicinchoninic acid (BCA) assay. Subsequently, hypothalamus Trp, 5-HT, and 5-HIAA were assessed using ELISA (MLBIO Co., Ltd. Shanghai, China).

### 2.6. Tryptophan-targeted metabolomics analysis

Serum from 42 days-old bird were sent to the metabolomics core at Zhongke New Life Biotechnology Co., Ltd. (Shanghai, China). A panel of 25 metabolites was assessed. The separation was performed on an ultra-performance liquid chromatography (UPLC) system (Agilent 1,290 Infinity UHPLC) on a C-18 column (Waters, CSH C18 1.7 μm, 2.1 mm × 100 mm column) by gradient elution. Positive switch mode was employed to perform the 5,500 QTRAP (AB SCIEX). MultiQuant or Analyst was employed for quantitative data processing.

### 2.7. 16S rDNA sequencing and analysis

16S rDNA were sequenced by Shanghai Zhongke New Life Biotechnology Co., Ltd. (Shanghai, China). Primer: 16S V3–V4: 341F-806R. All specific manipulations were performed as previous studies (23).

### 2.8. Statistical analysis

Data was analyzed using SPSS statistical software. Data for the TN and HS groups, the HS and HS + 0.18%Trp groups were analyzed using independent Students *t*-test. All data was expressed as the mean ± standard error of the mean (SEM). Differences were considered to be statistically significant at *p* < 0.05.

## 3. Results

### 3.1. Rectal temperature and serum hormones related to stress

**Figure 1:**
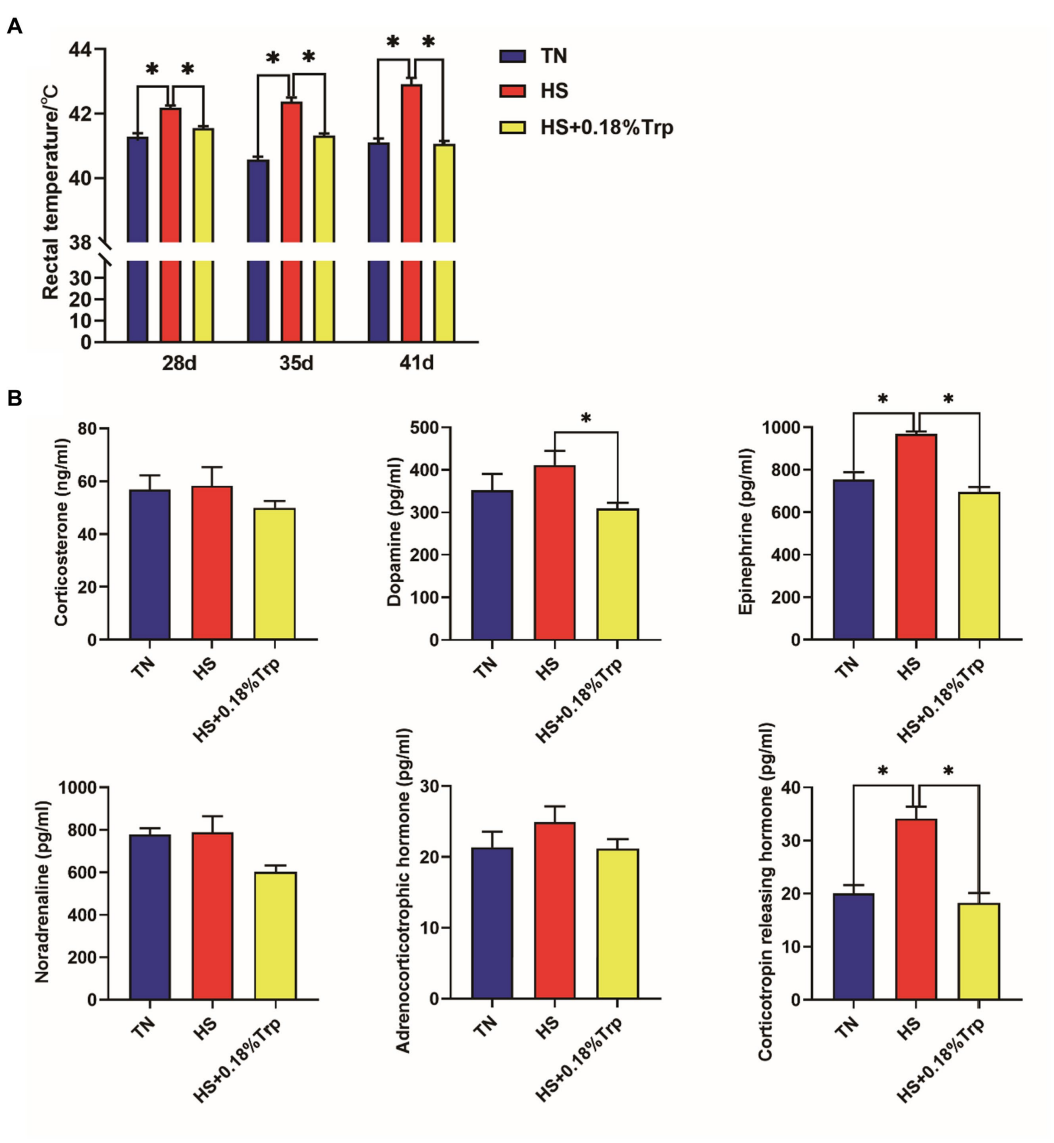
Effect of heat stress and dietary Trp supplementation on the rectal temperature **(A)** and serum hormones related to stress **(B)** of broilers. TN, chicks fed with a basal diet raised under thermoneutral conditions; HS, chicks fed with a basal diet raised under heat stress conditions; HS + 0.18%Trp, chicks fed with a basal diet supplemented with Trp by 0.18% raised under heat stress conditions. Mean values with * differ significantly (*p* < 0.05).

HS increased the rectal temperature of broilers on days 28, 35, and 41, as well as the levels of EPI and CRH in the serum (*p* < 0.05) as illustrated in Figure 1. In contrast, supplementation of Trp reversed all these changes in heat-stressed broilers and decreased the level of serum DA (*p* < 0.05). Additionally, no significant difference was observed among the three treatments in the levels of CORT, NOR, and ACTH in the serum of broilers (*p* > 0.05).

### 3.2. Immune organ index and serum IgA, IgG, and IgM levels

**Figure 2:**
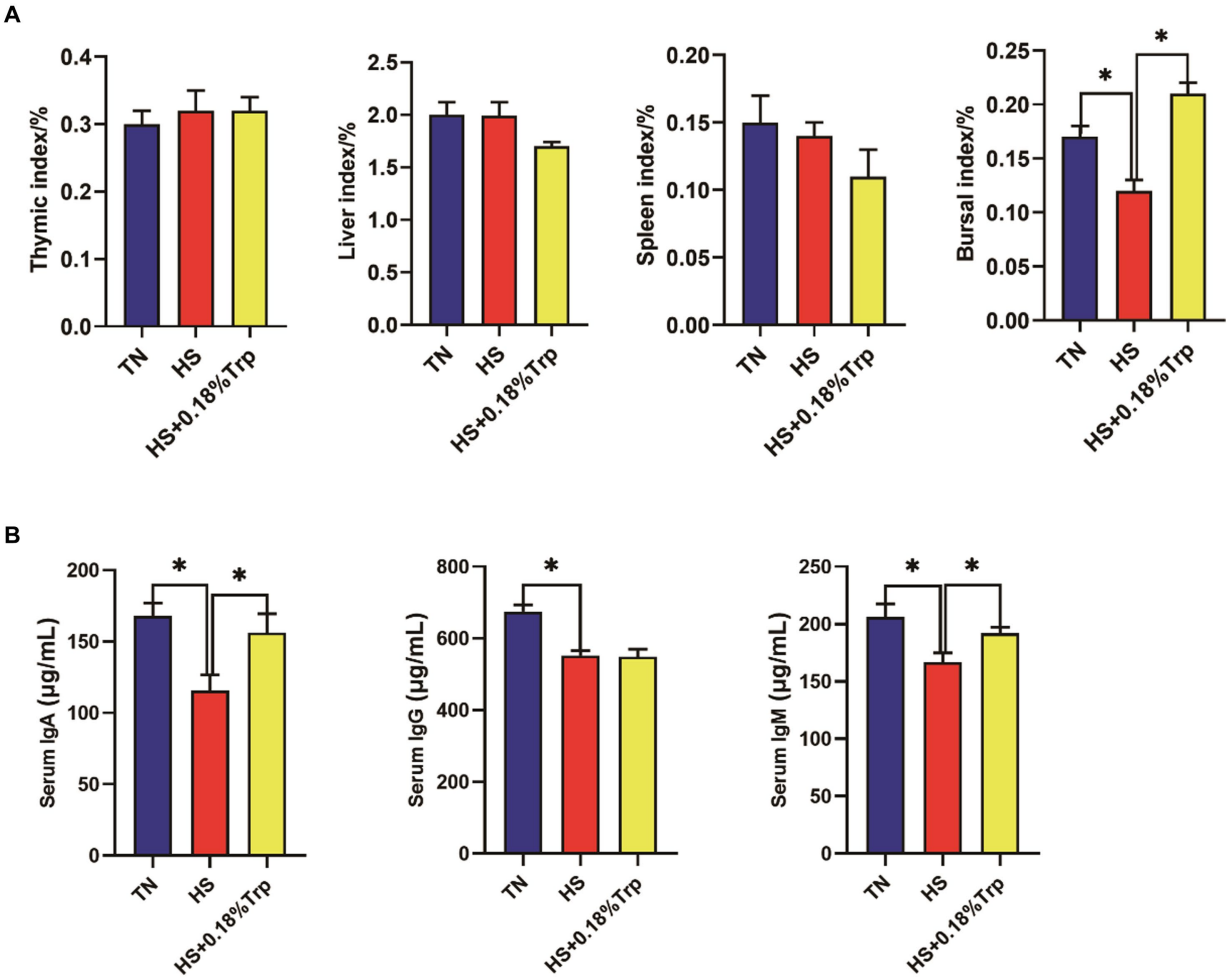
Effects of heat stress and dietary Trp supplementation on immune organ index and serum IgA, IgG, and IgM levels of broilers. TN, chicks fed with a basal diet raised under thermoneutral conditions; HS, chicks fed with a basal diet raised under heat stress conditions; HS + 0.18%Trp, chicks fed with a basal diet supplemented with Trp by 0.18% raised under heat stress conditions. Mean values with * differ significantly (*p* < 0.05).

As illustrated in Figure 2, HS significantly decreased the bursal index, serum IgA, IgG, and IgM levels in comparison to the TN group. Supplementation with Trp significantly increased the bursal index and the levels of serum IgA and IgM in comparison to the HS group.

### 3.3. Hypothalamic and liver tryptophan-degrading enzyme-associated genes expression

**Figure 3:**
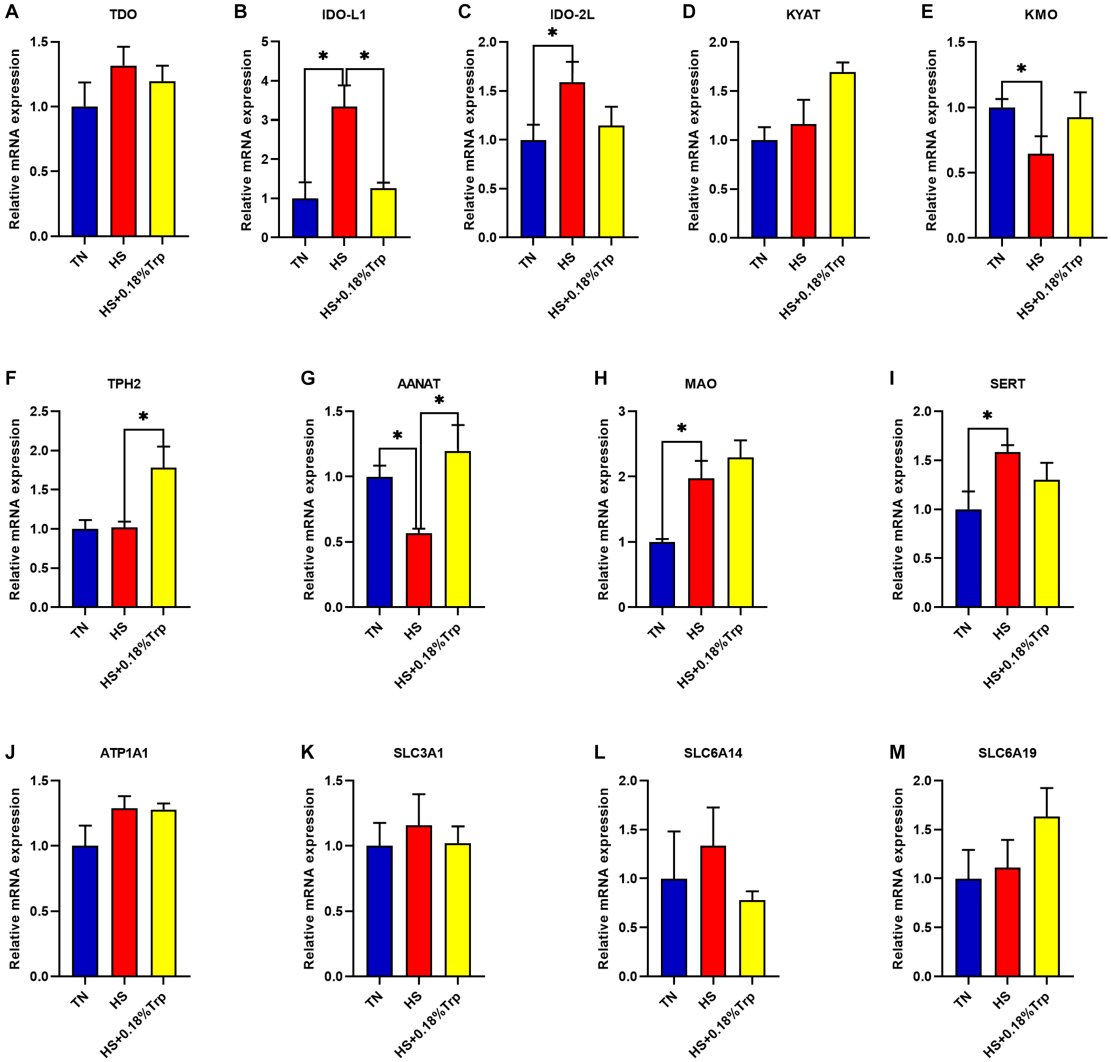
Effects of heat stress and dietary Trp supplementation on hypothalamic tryptophan-degrading enzyme-associated genes expression of broilers. TDO, Tryptophan 2,3-dioxygenase; IDO, Indoleamine2,3-dioxygenase; KYAT, Kynurenine aminotransferase; KMO, Kynurenine 3-monooxygenase; TPH2, Tryptophan hydroxylase 2; AANAT, Aryl alkylamine N-Acetyltransferase; MAO, Monoamine oxidase; SERT, Serotonin transporter; ATP1A1, ATPase Na+/K+ transporting subunit alpha 1; SLC3A1, solute carrier family 3 (amino acid transporter heavy chain), member 1; SLC6A14, solute carrier family 6 (amino acid transporter), member 14; SLC6A19, solute carrier family 6 (amino acid transporter), member 19. TN, chicks fed with a basal diet raised under thermoneutral conditions; HS, chicks fed with a basal diet raised under heat stress conditions; HS + 0.18%Trp, chicks fed with a basal diet supplemented with Trp by 0.18% raised under heat stress conditions. Mean values with * differ significantly (*p* < 0.05).

As illustrated in Figure 3, HS significantly increased the relative mRNA expression of IDO-L1, IDO-2 L, monoamine oxidase (MAO), and serotonin transporter (SERT) (*p* < 0.05), decreased the relative mRNA expression of kynurenine 3-monooxygenase (KMO) and aryl alkylamine N-Acetyltransferase (AANAT) (*p* < 0.05), and had no significant effect on the relative mRNA expression of TDO, kynurenine aminotransferase (KYAT), TPH2, ATPase Na+/K+ transporting subunit alpha 1 (ATP1A1), solute carrier family 3 member 1 (SLC3A1), SLC6A14, and SLC6A19 (*p* > 0.05) in the hypothalamus of broilers in comparison to the TN group. Supplementation with Trp considerably decreased the relative mRNA expression of IDO-L1 (*p* < 0.05) and increased the relative mRNA expression of TPH2 and AANAT (*p* < 0.05) but had no significant effect on the mRNA expression of TDO, IDO-2 L, KYAT, KMO, MAO, SERT, ATP1A1, SLC3A1, SLC6A14, and SLC6A19 (*p* > 0.05) in the hypothalamus of heat-stressed broilers in comparison to the HS group.

**Figure 4:**
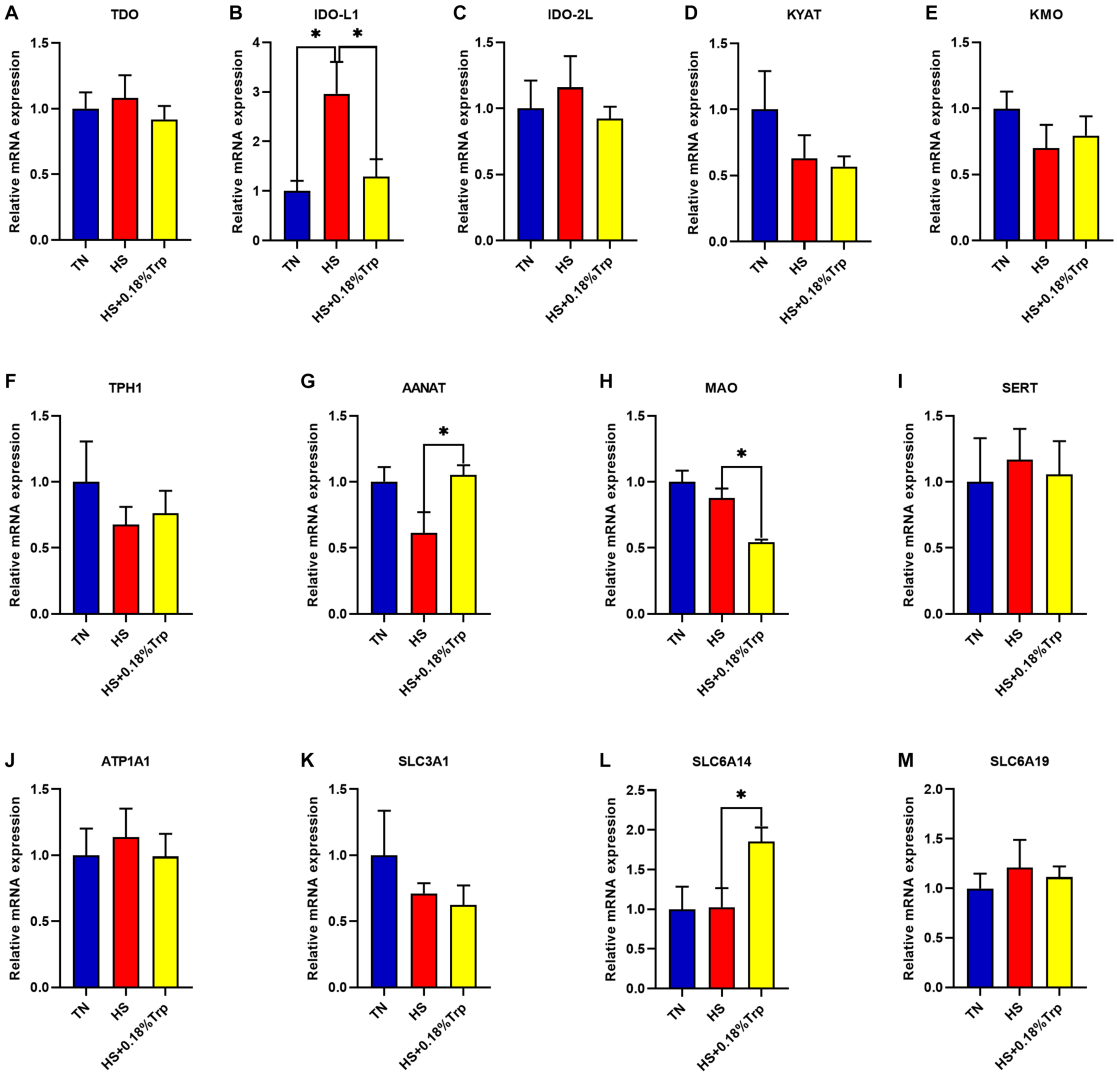
Effects of heat stress and dietary Trp supplementation on liver tryptophan-degrading enzyme-associated genes expression of broilers. TDO, Tryptophan 2,3-dioxygenase; IDO, Indoleamine 2,3-dioxygenase; KYAT, Kynurenine aminotransferase; KMO, Kynurenine 3-monooxygenase; TPH1, Tryptophan hydroxylase 1; AANAT, Aryl alkylamine N-Acetyltransferase; MAO, Monoamine oxidase; SERT, Serotonin transporter; ATP1A1, ATPase Na+/K+ transporting subunit alpha 1; SLC3A1, solute carrier family 3 (amino acid transporter heavy chain), member 1; SLC6A14, solute carrier family 6 (amino acid transporter), member 14; SLC6A19, solute carrier family 6 (amino acid transporter), member 19. TN, chicks fed with a basal diet raised under thermoneutral conditions; HS, chicks fed with a basal diet raised under heat stress conditions; HS + 0.18%Trp, chicks fed with a basal diet supplemented with Trp by 0.18% raised under heat stress conditions. Mean values with * differ significantly (*p* < 0.05).

As illustrated in Figure 4, HS significantly increased the relative mRNA expression of IDO-L1 (*p* < 0.05) in the liver in comparison to the TN group. Supplementation with Trp significantly decreased the relative mRNA expression of IDO-L1 and MAO (*p* < 0.05) and increased the relative mRNA expression of AANAT and SLC6A14 (*p* < 0.05) in the liver in comparison to the HS group. In the liver of each treatment group, there was no significant difference in the relative mRNA expression of TDO, IDO-2 L, KYAT, KMO, TPH1, SERT, ATP1A1, SLC3A1, and SLC6A19 (*p* > 0.05).

### 3.4. Hypothalamic and liver Trp metabolite levels

**Figure 5:**
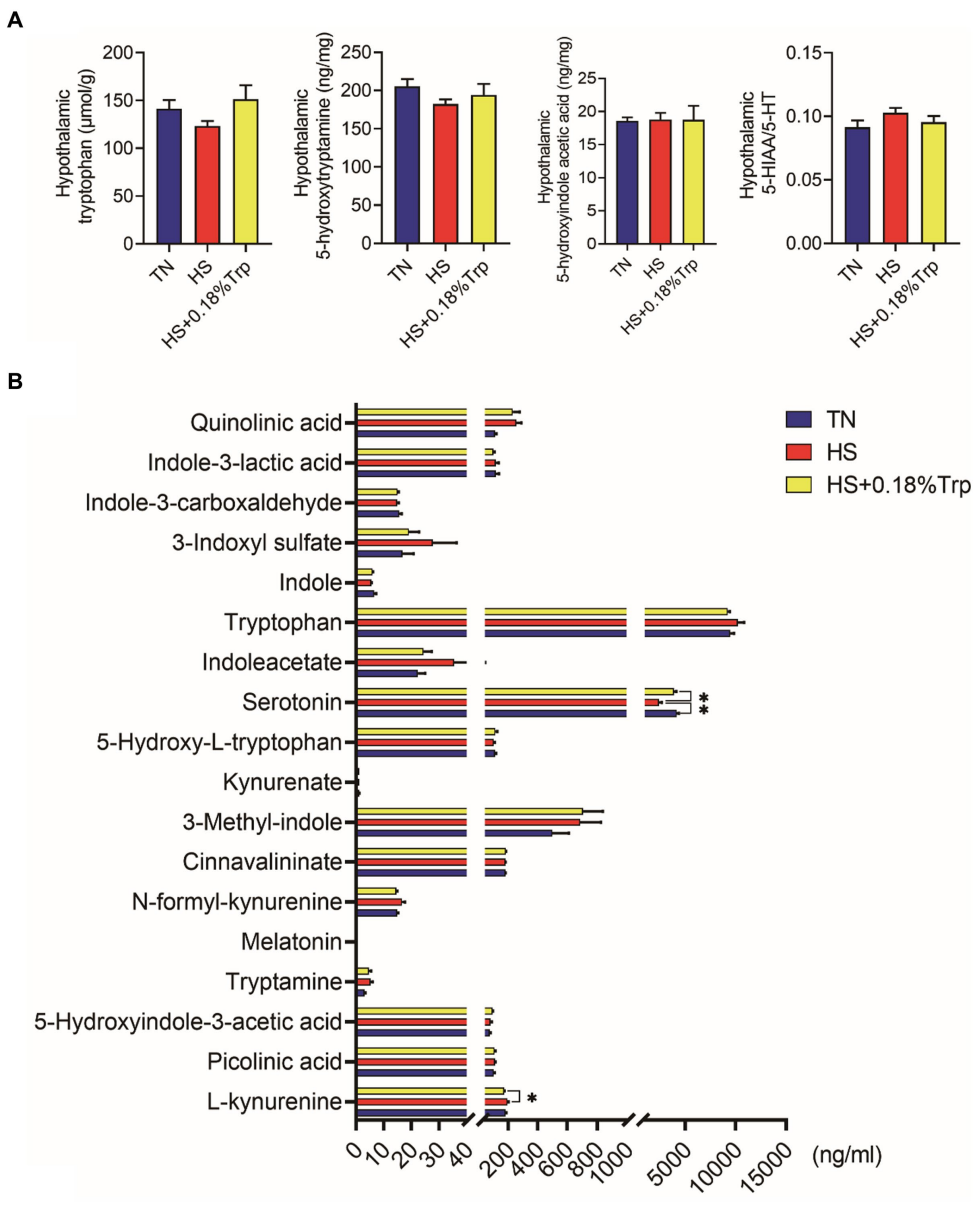
Trp metabolite contents in hypothalamic **(A)** and serum **(B)**. TN, chicks fed with a basal diet raised under thermoneutral conditions; HS, chicks fed with a basal diet raised under heat stress conditions; HS + 0.18%Trp, chicks fed with a basal diet supplemented with Trp by 0.18% raised under heat stress conditions. Mean values with * differ significantly (*p* < 0.05).

No significant difference was observed among treatments in hypothalamic Trp metabolites (*p* > 0.05), as illustrated in Figure 5A.

HS significantly decreased the level of serum 5-HT in comparison to the TN group (*p* < 0.05), as illustrated in Figure 5B. Compared with the HS group, supplementation with Trp considerably increased the serum 5-HT level (*p* < 0.05), and decreased the serum L-kynurenine levels (*p* < 0.05) of heat-stressed broilers.

### 3.5. Cecal microbiota composition

**Figure 6:**
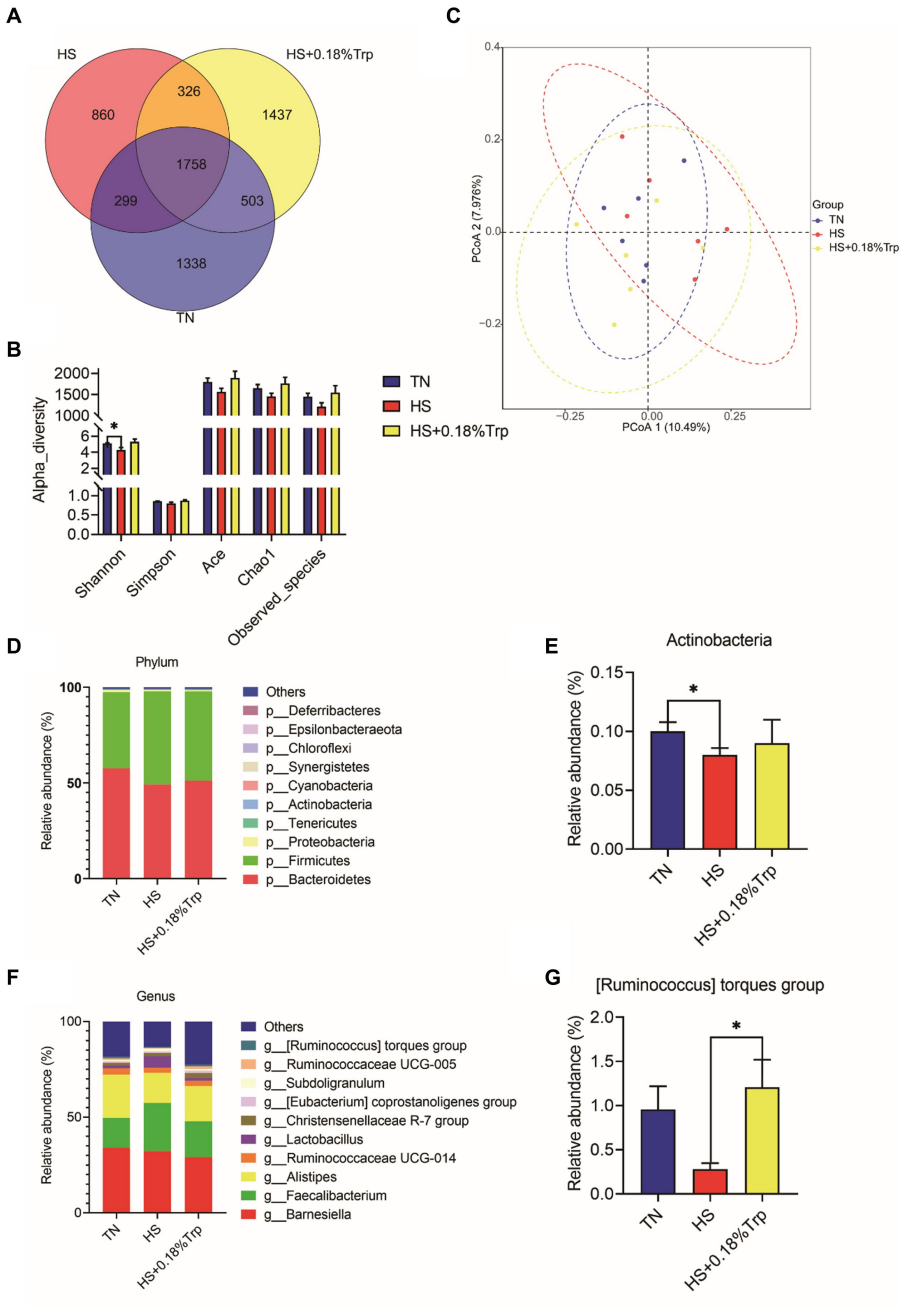
Effect of heat stress and dietary Trp supplementation on the caecal content microbiota composition. **(A)** Venn diagram of OTUs, **(B)** α-diversity index, **(C)** PCoA plot, (**D, E**) Microbiota compositions at the phylum level, and (**F, G**) Microbiota compositions at the genus level. TN, chicks fed with a basal diet raised under thermoneutral conditions; HS, chicks fed with a basal diet raised under heat stress conditions; HS + 0.18%Trp, chicks fed with a basal diet supplemented with Trp by 0.18% raised under heat stress conditions. Mean values with * differ significantly (*p* < 0.05).

Using 16S rDNA sequencing, the cecal content was analyzed 1,338 unique operational taxonomic units (OTUs) were identified in the TN group, 860 in the HS group, and 1,437 in the HS + 0.18%Trp group, with the same OTU number of 1758 (Figure 6A). Alpha diversity was applied for analyzing the complexity of species diversity for a sample through multiple indexes, including Observed species, Chao, Ace, Shannon, and Simpson. With the exception of Shannon, which was decreased (*p* < 0.05) by HS, dietary Trp supplementation and HS had no significant impact on the bacterial richness of cecum in broiler chickens (Figure 6B). According to the matrix of beta diversity distance, principal coordinate analysis (PCoA) exhibited that bacterial communities differed significantly in their composition among the samples (Figure 6C). The phylum and genus levels of the cecal microbiome were examined to further explore the effect of HS and dietary Trp supplementation on the cecal microbiota composition. The measured OTU was classified based on a comparison with the corresponding database. The most prevalent phyla in the cecal-content microbiota of broilers were Bacteroidetes, Firmicutes, and Proteobacteria. Compared with the TN group, HS significantly decreased the relative abundance of Actinobacteria (*p* < 0.05); however, supplementation with Trp significantly increased the relative abundance of *[Ruminococcus] torques group* in the cecal content in comparison to the HS group (*p* < 0.05).

## 4. Discussion

The important indexes to determine whether animals are in a stressed state include rectal temperature, blood pH, respiratory rate, etc. (24–26). The rectal temperature of broilers was determined on days 28, 35, and 41 of HS to assess the effect of persistent HS. According to the results, HS significantly increased the rectal temperature of broilers, which was in accordance with the results of Vesco (27). Dietary Trp supplementation significantly reduced the rectal temperature of broilers with HS, and similar results found that intravenous injection of Trp reduced the increase of rectal temperature caused by acute HS in the steers by increasing the level of brain 5-HT (28). In a previous study, we found that 0.18% Trp supplementation decreased the rectal temperature in broilers under the condition of acute HS (29). This finding is consistent with previous observations that dietary Trp supplementation alleviated the increase of body temperature caused by HS in broilers.

When the animal is stimulated by the external environment, the ANS and HPA axis become excited, resulting in sympathetic nerve excitation and stimulating the adrenal medulla to release EPI, NOR, and DA to maintain the endocrine stability of the body (30). In this experiment, serum EPI and CRH levels of broilers in the HS group were significantly increased in comparison to the TN group, indicating that HS activated the sympathetic nervous system while the body was under stress. Baxter Mikayla found that HS significantly increased the serum CORT level of broilers, but this phenomenon was not observed in this experiment, which might be a result of the varying degrees of HS caused by different temperatures and time factors (31). Dietary Trp supplementation significantly decreased the serum EPI and CRH contents of heat-stressed broilers. In a previous study, we also found that 0.18% Trp supplementation decreased serum DA, adrenaline (Adr), NA, CRH levels in broilers under the condition of acute HS (29).Yue demonstrated in a similar study that dietary Trp supplementation significantly reduced the increase of serum CORT, EPI, and NOR levels of broilers caused by chronic stress and alleviated the stress state of the body (12). Liu demonstrated that dietary Trp significantly decreased plasma CORT, EPI, NOR, and hypothalamic 5-HT levels and reduced the weaning stress of piglets (32). The results suggested that dietary Trp supplementation significantly decreased the serum stress-related hormone levels of heat-stressed broilers.

The immune organ index reflects the immune function of the organism. Studies have demonstrated that the development of immune organs is inhibited, and the indexes of the thymus, spleen, and bursa of Fabricius are decreased when animals are subjected to high-temperature environments (33, 34). In this study, it was demonstrated that HS significantly decreased the bursae index of broilers, which was in accordance with the results of Calefi (33) and Chang (34), indicating that HS affected the bursae development of broilers. This study indicated that dietary Trp supplementation significantly increased the bursa of Fabricius index of heat-stressed broilers, indicating that dietary Trp supplementation reduces immune organ dysplasia of broilers caused by HS.

Immunoglobulins are an integral component of the adaptive immune system, and their changes reflect the state of humoral immunity of the body (35). Studies have demonstrated that under HS, the serum levels of IgA, IgG, and IgM of broilers decreased (36, 37), and this phenomenon was consistently observed in this study, indicating that HS reduced the immunity of broilers. Dietary Trp supplementation significantly increased the serum IgA and IgM levels of broilers with HS and enhanced the immunity of the body.

Approximately 1–2% of Trp is metabolized through the serotonin pathway, and converted into 5-HT and melatonin by TPH and aromatic amino acid decarboxylase. 5-HT is involved in regulating the stress response of the body through the HPA axis (38). HS significantly increased the expression of hypothalamus MAO and serotonin transporter SERT, decreased serum serotonin content and the expression of hypothalamic AANAT of broilers in comparison to the TN group, indicating that HS might increase the 5-HT-5-HIAA metabolic pathway, convert 5-HT into 5-HIAA, and excrete from the kidneys, resulting in a decrease in 5-HT content and a weakening of 5-HT to melatonin metabolism pathway, which is consistent with the study results of Yue (12). In addition, our previous results demonstrated that HS significantly reduced the average daily feed intake (ADFI) of broilers and the lack of Trp intake in broilers resulting from the low average daily feed intake might be another important reason in the decrease of serum 5-HT level (39). In animal models of colitis, Trp deficiency caused by decreased feed intake has emerged as one of the major aggravating factors of chronic inflammatory susceptibility (40, 41). IDO is particularly active in the immune system and mucosal epithelial tissues and is significantly expressed under immune stress conditions (42). In this study, the catabolic enhancement of IDO on Trp under HS might be the factor leading to further reduction of 5-HT levels (43–45). Over 95% of available Trp has degraded through the KYN pathway, relatively minimal changes in the activity of this pathway might have substantial effects on the flow of Trp into the brain (46). Therefore, the reduction of Trp influx into the brain as a result of the activation of the KYN metabolic pathway caused by HS might be one of the potential factors for HPA disorders (47, 48).

Although dietary Trp supplementation did not significantly increase the level of serum Trp, it significantly increased the expression of tryptophan transporter SLC6A14 in the liver, the absorption and utilization of Trp (12), the level of 5-HT content, and maintained gut homeostasis (49, 50). Dietary Trp supplementation significantly increased hypothalamus TPH2 expression of heat-stressed broilers, enhanced the 5-HT metabolic pathway, and significantly increased serum 5-HT content, which was in accordance with the study results of Liu (51) and Bello (52). In addition, dietary Trp significantly increased the expression of the hypothalamus and liver AANAT of broilers under HS and increased the 5-HT-melatonin metabolic pathway, which could not be detected as a result of the low serum melatonin content of broilers. Studies have demonstrated that dietary Trp supplementation reduced the concentration of plasma CORT, thus, alleviating the stress response of pigs (32). Trp decreased rectal temperature in steers under acute HS by increasing brain 5-HT (28). The above analysis shows that dietary Trp supplementation enhanced the 5-HT metabolic pathway, decreased the 5-HT-5-HIAA metabolic pathway, increased the synthesis of 5-HT, reduced the contents of EPI and CRH, relieved HS of broilers, alleviated the disturbance of stress-related hormone levels caused by HS (53), reduced the levels of TDO and IDO further, and reduced the production L-kynurenine.

Furthermore, another metabolic pathway of Trp is the microbial pathway. According to PCoA, the microbial composition of cecum contents under HS was significantly different in comparison to the TN group, and HS significantly reduced the number of unique OTUs, Shannon index, and altered the gut microbiota composition and diversity (54). There was a notable increase in the relative abundance of Actinobacteria under HS. Actinobacteria, classified as gram-positive bacteria, play a role in metabolizing starch and starch-like polysaccharides and oligosaccharides to produce lactate and acetate (55). However, there is no conclusive evidence for the relationship between HS and the Actinobacteria. We speculate that the Actinobacteria may contribute to HS in broilers by increasing heat production in the cecal metabolism. Dietary Trp supplementation improved cecal microbial composition and significantly increased the relative abundance of [Ruminococcus] torques group. In humans, [Ruminococcus] torques group was found diminished and associated with anti-inflammatory activity in Crohn’s disease under the chronic immune-mediated inflammatory condition (56). In broilers, [Ruminococcus] torques group may be positively associated with growth performance, intestinal mucosal barrier and alleviating inflammation (57, 58). In addition, it has been reported that indole-producing bacteria prevent the growth and survival of non-indole-producing bacteria, such as Enterobacter, especially in Salmonella and Shigella (59), and might be one of the reasons why Trp supplementation changes the relative abundance of intestinal bacteria. However, as limited information on correlation between Trp and [Ruminococcus] torques group, future studies need to be undertaken to determine whether Trp induces the selection of the [Ruminococcus] torques group and to understand the role of the [Ruminococcus] torques group changes in the regulation of HS.

## 5. Conclusion

HS significantly increased the rectal temperature, decreased the level of serum 5-HT, and resulted in an increase of stress-related hormone levels, which in turn resulted in an abnormal KYN metabolic pathway and decreased immunity of broilers. Dietary Trp supplementation significantly decreased the rectal temperature, increased the level of serum 5-HT, decreased the secretion of stress-related hormones, inhibited the KYN metabolic pathway, reduced the L-kynurenine level, and enhanced the humoral immunity of heat-stressed broilers.

## Data availability statement

The datasets presented in this study can be found in online repositories. The names of the repository/repositories and accession number(s) can be found in the article/Supplementary material.

## Ethics statement

The animal study was approved by Laboratory Animal Ethics Committee of Jiangxi Agricultural University. The study was conducted in accordance with the local legislation and institutional requirements.

## Author contributions

GL and QL conceived, designed the experiments, and wrote the manuscript. QL, JO, HZ, GL, and CD collected samples and performed the experiments. QL, JY, and GL performed the analysis. GL and HZ directed the analyses and revised the manuscript. All authors contributed to the article and approved the submitted version.
