# Supplementary material for: Effects of dietary tryptophan supplementation on rectal temperature, humoral immunity, and cecal microflora composition of heat-stressed broilers
Source: Front Vet Sci. 2023 Sep 28;10:1247260. doi: 10.3389/fvets.2023.1247260 (PMC10572358; doi:10.3389/fvets.2023.1247260)
Supplement: Supplementary file 3 [file Data_Sheet_1.ZIP › 03_Community/krona/krona.html]

Javascript must be enabled to view this page.

magnitude
magnitudeUnassigned

HS\_1
HS\_2
HS\_3
HS\_4
HS\_5
HS\_6
HS\_Trp\_1
HS\_Trp\_2
HS\_Trp\_3
HS\_Trp\_4
HS\_Trp\_5
HS\_Trp\_6
TN\_1
TN\_2
TN\_3
TN\_4
TN\_5
TN\_6

583632888659933468108248042346271135748767928481914602035237326343995345707852825019635502

583572888659933468108248042346271135748767928481884602035237326343995345707852815019635502
405278531517963341141401561789158532348471334224645292

000000000000000002

000000000000000002

000000000000000002

000000000000000002

341011131015086232
000000000000020000

020000000000000000

020000000000000000

020000000000000000

020000000000000000

020000000000000000

321011131015066232

010000000011052020

010000000011052020

010000000011052020

000000000000000101
311011131004014212

110011001003003001

100000000000002000

010011001003001001

201000130001011110
100000110000011100

101000020001000010

101000020001000010

000000200000000000

000000200000000000

000000200000000000

000000200000000000

000000200000000000

000000200000000000

400000000000000000

200000000000000000

200000000000000000

200000000000000000

200000000000000000

200000000000000000

200000000000000000

200000000000000000

200000000000000000

200000000000000000

200000000000000000

151131700023021510

151131700023021510

111020000010021510

111020000010021510

111020000010021510

040111700013000000

040111700013000000

040111700013000000

040111700013000000

010100000010000010

010100000010000010

000100000010000000

000100000010000000

010000000000000010

010000000000000010

010000000000000010

010000000000000010

042120230142022032

042120230142022032

042120230142022032

042120230142022032

041120120132022031
042120120142022032

001000000010000001

000000110000000000

100001000001002000

100001000001002000

100001000001002000

100001000001002000

100001000001002000

100001000001002000

11130210009220000002

11130210009220000002

11130210009220000002

11130210009220000002
030000000000000000

01100000009000000001

01100000009000000001

100210000220000001

100210000220000001

131310281331213212121427632676855

113124103019111613721255351

000044000000441000

000044000000001000

000000000000440000

000000000000440000

000000000000440000

212320010102334321

212320010102334321

000100010102222111

000100010102222111

212220000000112210

212220000000112210

11100142618111511014202150

1828243114105111204217154

9003000130012180100
1828243114105111204217154

11123111551114816834

11123111551114816834

11123111551114816834

81719000220094160220

81719000220094160220

81719000220094160220

002000000000000050

002000000000000050

002000000000000050

9411718911711362106136545178692721111072122756996

9411718911711362106136545178692721111072122756996
020000000000000000

101000200100003021

101000200100003021

101000200100003021

0799731029121712065161

0799731029121712065161

010010301100031030
0799731029121712065161

069963729111612034131

686495675816535123480371504368581152235
921061771081045392107531155672691071012032746094

000000000433000000

000000000433000000

000000000433000000

2240753343363753283692510361301341393356

2240753343363753283692510361301341393356

2240753343363753283692510361301341393356

2278312314221333112053

2278312314221333112053

2278312314221333112053

121026100211101010

121026100211101010

121026100211101010

121026100211101010

001000102300300000

000000100300300000

000000100300300000

000000100300300000

001000002000000000

001000002000000000

001000002000000000

010010000200003000
6423655582702851923963615062512583244323673201778414624

123199144471081558018513891631211048494778171260

000000000000200000

000000000000200000

000000000000200000

000000000000200000

000011010002012110

000011010002012110

000011010002012110

000011010002012110

2000000030014000010

200000003000000000

200000003000000000

200000003000000000

0000000000014000010

0000000000014000010

2516882581162431341036822020712120209

0000700200011200215
2516882581162431341036822020712120209

000000000000000505
2516882511162429341035811820712118194

2516882511162429341035811820707118189

000000000002000000

000000000002000000

000000000002000000

000000000002000000

551563121222227138101673526172740391736

000001010000000011

000001010000000011

109331036697812711839
51150241620152613296632924102238261233

603030061000036000

603030061000036000

000002001101000400

000002001101000400

239137471677191916671512621

239137471677191916671512621

011010000000111000

310100130310033021

310100130310033021

212700031110783003000011

212700001110783000000011

000003000003000000

533400100102101100

200100000201011101

200100000201011101

001020100010000000

001020100010000000

123222011122411221

020101001001210010

020101001001210010

103121010121201211

103121010121201211

210000030100310210

210000030100310210

210000030100310210

024304114240031911

011001011100010400

011001011100010400

013303103140021511

013303103140021511

110000000000000000

110000000000000000

110000000000000000

4022181920152492919153523431263115

271814167121632117102502924252112

67030090633130716121
19162112192165625020820174

200510016232072402

119231101400100651051

82645861510000816248
8212551071511300916548

006102100130010300

000001000110000000

1344313386825102571103

1344313386825102571103
422142346145027020

922291522115230183

153561562136121010

021201330016120000

010101330012020000

010101330012020000

000100000001100000

011000000003000000

132360232120001010

000130000000001000

000010020000000010
022010130110000010

022000110110000000

110220102010000000

110220102010000000

004010000000000000

004010000000000000

004010000000000000

004010000000000000

434639442729595057405053225237454674
000000000000000020

001010010001000000

001010010001000000

001010010001000000

001010010001000000

110012020010010111

110012020010010111

110012020010010111
000001010000000010

110011010010010101

032210101511035110

032210101511035110

020000101311022100

012210000200013010

012210000200013010

14000000011000002019

14000000011000002019
13000000000000002019

100000001100000000

100000001100000000

000000000000000200

000000000000000200

000000000000000200

000000000000000200

101000210011000100

101000210011000100

011000101000200000

011000101000200000

011000101000200000

011000101000200000

010202002021100030

010202002021100030

010202002021100030

010202002021100030

0221351573161110120485

0221351573161110120485
00264116290010010342

010210013000000002

010210013000000002

000200102000100001

000200102000100001

010304002110010140

010304002110010140

916101593192316222726142417141021

000000002000000000
916101593192316222726142417141021

7737511314914191981881106

010000000000010010

010000000000010010

011000020311011001
264120452645652367

253120432334641366

000002112112000021

023720131130007017

023720131130007017

003000000010000000

003000000010000000

003000000010000000

003000000010000000

3592522115121050379

349042305111050178

349042305111050178

349042305111050178

010100000010000201

010100000010000201

010100000010000201

0000001800000000000

000110010000000000

601300121132044434

000000010021000000

000000010021000000

000000010021000000

601300111111044434

201200011111031112

201200011111031112

400100100000013322

400100100000013322

9179755717149111041311131115
000000020000000000

020200253114001212

020200253114001212

8863142684743834710
231301125142132333

013001022000000011

013001022000000011

642012121332251136

130000100101003411

020000000100000011

020000000100000011

110000100001003400

110000100001003400

000010000110000000

000010000110000000

000010000110000000

043231243221154322
000000000000020000

012111233001013310

000020000200000000

031100010020121012

031100010020121012

000000000000000300
4761193751791498257126311118145150306231186955197290

002100000000000000

002100000000000000

002100000000000000

110000000000001000

110000000000001000

110000000000001000

110000000000001000

4741183731771488256124309117144150306230184951197290

2174865440192477004315183140362117
4741183731771488256124309117144150306230184951197290

010000000023000002423

02031213201100203300

010200100100000100

00000013001000000000

010112100000203200

109441122668242648140058391621651

109441122668242648140058391621651

1482319610479311174183102121107951081794839599
1482319610479111174182102121107951081794839399

000002001000000020

100110122110011100

100110122110011100

100110122110011100

100110122110011100

21971362756153714613533146429138249667149
340311640319501174395825215269181771800937954291931030414597140181830018401125951704921321

012000202000000000
334827178171231645856676148621769215353325702873499151382413758170401784592631620219124

334777164171111644756675148591768315353325292871399121381113752170281783892111618919114
2842074914784122745263661157647151472521739438297583320

41411467102032375159161815185
3335443333192749105832333293757546232

000001000200230000
000000000000020000

000001000200010000

000000000000200000

213000010111000200

213000010111000200

23152825237102764361311181434313024
111122172056205232118131128262322

12468324712423433562

000000000000103010

4524447197460456143

4524447197460456143

200000001600003010

000000000500000000

000000000500000000

200000001100003010

200000001100003010

769435116462112049909727141925585346639844940871880704563
184110872390124030881605251515404059200510061822158925312102176325531120

100201602504133510
000000000002000000

100201602502133510

001001001100000011

001001001100000011

1210162270113721818739181407

000040023813100200

1210162230113518017438181207

127271184253087153412012

127271184253087153412012

30311422572045797517375010515079227

9210023006114313110

5882360971797917720226044861801831301018411078

5882360971797917720226044861801831301018411078

79148691218481921540702616

6104151076518141369114612

6104151076518141369114612

000000000100101021
460250403061411840173

460250403051401830152

20147831583335471381512178784356796419
20147831583333471371512178784356796419

000000201000000000

1251232254031110101

1251232254031110101

3153208006000460701

020200000000000000
11452118574128455737498158262445686245

11450118554128455737498158262445686245

117297136867852015521801056642141260104535707377598121
120298141868092035631801062653142265105539713377603123

000020000000000000

315022211061115146052

1201183029030111571

1792379612182969312816143264205431

110010136203042003

000000030400300000
11616213311306715366

013140601904013344

103022700002402022

100000001000201000

100000001000201000

100150000301023001
000000000301020001

100150000000003000

44046190209419383387323928413239860634646959745
44046190209419383387323928413239860634646659745

000000000000000300

112482404715215624182707290651475541103189124

112482404715215624182707290651475541103189124

001010020100400200

001010102131000134
134412152101081051013177

000020000100000010

020100000010000001

113391428840051012132

101000012002301100
201210012412316112

100010000010000000

000000000000004000

000200000400011012

000072000200013302

000072000200013302

756309125615664082845511091278719174921029438560326767572879
110110206123213253

519185902124621417541985118991431336819138368121338342668
754303125215604052835421087277419124851025433552321764560872

001001001010001101

009402030403010000

25241010218178136243111696284011

100000001000002112

000010010010020120

6221405726161357113711520233238544417

71231200266090613921070

1378329322816288841615182551061402487190351123172

300311304626005011

154521747451372174
012110022000101000

142411725451271174

311644532160073810241

311644532160073810241

000021010001000000

311642431160063810241

000000001010000400

000000001010000400

000000001010000400

292210116035121124728725226

292210116035121124728725226

292210116035121124728725226

78221132594639220283254718652025
1181132921276336236592708498789422231677499127343

1111052651166034177546669278509119726959434107317

1111052651166034177546669278509119726959434107317

005000000000000001

005000000000000001

373464261017454214438630326440932433
002613613002025815

310001206000323200

000000000000300000

310001206000023200

0210000019800252110

0210000019800252110

34316120913374111630628275530822228
33274917811323210327621235121712025

010000130000001100

131231246133074481023

1600411300004112002

1500010200001100001

1500010200001100001

100401100003012001

100401100003012001

001000000001010000

170251152102416317475615814011579141673598226543574740111
3214975981432477317781080522541092418519833719

132401110102120510

132401110102120510

0331216214410421216004

0331216214410421216004

601300311439207110

601300311439207110

44132445351310378145951121712335228346

44132445351310378145951121712335228346

768211135498111142373832876517731510131528629575

768211135498111142373832876517731510131528629575

8148015707245101020251326

8148015707245101020251326

300000226186600718773701

300000226186600718773701

010000010000000000

010000010000000000

20057291185120823368671836989311723378441529106318503333104419801152
30215511712403119245249312433129611150922099219198013962210312125171416851531150116267

7972710819713211462221945131436
000000000000000002

7972710819713211462221945131434

000000003400000000
50147111882981168712689

000000002000000000

001000000200000002

50137111882421168712687

200200002000002000

200200002000002000

0106207214503144611
0105002112103132000

000120512400012611

300000000000000000

300000000000000000

34127104023477215101710830442

000000000000030000

34127104023477215101710530442

2418611527247715945748971377236585770716546533953315742569167121549662411550
1641911779167611385716629547778010417927130147

202100110714041060

5435501841124038626178275111663218487660011393

239661098707868954557094127198650173631596951805184563053995656922637511310

69159152714162347141914261211
6854139994943713622952293957128139968846

139672809381110111126040613

010011001000200110

433552581826341498242023264819244020

50042920010460351005270

105000702502406022

3729597629347138109212890646781536648
12581552126222620815137107

242144511926562772172164494059364227

100502100003321040

03755425152134108101014

128425464040312834112916732551461987125
8718312013106302906236419993

02348352123317211533172

310141012221319185840126151384019

1101023191135371851

213223670526215521383032

13118011621820610452211
1520252522431134411612182417151432

1417141722315112696682012131221

000000000000005000

000000000000005000

1019140110471147413974813656425798154418453168273224466
101811881765361332271034504459612836638816525425445

11159141238002221012

0428011429451533011322450631919819

000001201102000000

017600000103000000000

010000000100000100

010000000100000100

000040340000011000
000030310000001000

000010030000010000

1727825609766100721054226924308111

1727825609766100721054226924308111

4042011137024323224

810341203745118030

200100000231102010

610241203514016020

4016043038311822120
401200301131120120

0004040007001702000

000000002000000000

2010000011022001060

2010000011022001060

218138314175249841423945123831287882204360135153164
6001170426299108814614980595255139127299624568167175233

378100511012383262741143216826318917405205312268

000000000000002000

0112020003000060000

4160150005005091101

231937526125742597281157692965137858
179488678222114112391333812916110530621517033828

98114923169848015730785104342011153225220

5818030001159333027635110

2444451501646459630648297028262325
004000007300028100

2444051501646458927648296820252325

611414163221311622228

611414163221311622228

1114122714292072803824100310433934988387703189222505279996622117
778992913782523647827756305375022844711461417452774499704

2194225094810053181319248113554705361532781279

117193266342180124158156789190462012762976019385134

88341134101639322140814671014646175223
11854213610175109231471014691065152175324

32120121617202556011

41614790198107933704724571913254721834481233156390138
2076969180717336324936139290373139344792136382110

000000000004100000

209782118362072315215235954310444120828

4779302925114951824595
1367200310230354213

020000024302101000

321210241161711319382

001100002001330000

001100002001330000

22277151348301101251731131511147
000000002000000000

22277151348301081251731131511147

6131610391856171039164228622982711
10152014402376191261111447287970153615

42441520223208501741794

113012236000411011

113012236000411011

7044764016627812811017125310348971941306466
51462414211514133106451

6004601019163941069405

000010000000013000

59417230135267626881401988644861131225960

020000002020003000

70173713523232625142575328323169
2112737138732829321627710338423178

002033022202006000

1418002031520054106

000000000000000003

000000200000000000

000000200000000000

75161051001797317014524895462091523712418284105
203712437105315133

407122111393001116063

69169592176691551292329146204138351131727599

182806000796801010516661
000000002000000000

1711060002704091114061

11700000524401942600

1153101091772519511961511104
901302306141333212

112552657004424631

000000201000000100

100012111110010100

04024351101706208161

68761747839964854235431969689975951228
6256163703787404013230135646605670615

618882781499851320391623511

020001000000000000

003001004510203212

5131011137039213136127521310

5131011137039213136127521310

5131011137039213136127521310

5131011137039213136127521310

1121481171526211519636450221781680331086132224

1121481171526211519636450221781680331086132224
000000126000010420

1121481171526211419616390221781679331082130224
002000050000000000

1121481151526211419566390221781679331082130224
000000026004020111

1121481151526211419546330221741677331081129223

2508944186482431525115928039971632314018129716218805331574
003000030000020036

524112289951355713182763297730
020000000000000000

618323634426661291011104

0101400115310002130

0101400115310002130

0000000014100000000

0000000014100000000

5161823337151096954

5161823337151096954

112040000000002110

112040000000002110

000100000100200010

0010000100000080460

0000000000000000460

001000010000000000

001000010000000000

000000000000008000

000000000000008000

45218534142417061845151926
282611311317064183419

0000000021000000000

0000000021000000000

000410010000001210
171924210300000142712157

171920110200000142610147

100002045000000320

100002045000000320

000000035000000000

100002010000000320

198890318497963062421542643942156218400732689918514531538
43151100600116001417

1100000000001003231160

000000000000001315150

000000000000001315150

110000000000100191610

110000000000100191610

024000000000000000
179889618467812552371502483932149217393712595818114111515

000000000000001200

000000000000001200

12787101717663123174591943538116151349581713217292961395
179889418427812552371502483932149217393712595718094111515

000000002000000000

313101113451000102

000000030000000000

0000000000000011130

1713516110955523229717521162727267

1133747167333111142623199965

000000000000002200

0013304422031030937232

000000000000002600

21167336401115912550017214

2000401103001103341

200010000001000111

100010000001000101

100000000000000010

0000391103000103230

010700000411010012

010700000300010002

010700000300010002

000000000111000010

23231033161303026673

22221033140103026660
23231033161303026673

010100021200000013

16636261129679139174267607263122130149145337270115250

16636261129679139174267607263122130149145337270115250

26611945320124358141271211717418
16636261129679139174267607263122130149145337270115250

0110026000220000001

011000000220000001

0000026000000000000

7023211045085532414

7023211045085532414

000070000000000000
0016391013030220000200

0016384013030220000200

9640312711211022102

9640312711211022102

5231001712405033214
4111966519395619428166126210281389344138

36989601939551832666112579678359143125

004400043100600009

7910144452076781626489914535361201413476
456692214612233730715518111579361833

3206415534632152292323248381041234

24118842271061340112147

000005430000021002

000000002300000000

000000000100030000

000000000100030000

000000011000012010

000000011000012010

2221901203900052614109

200032000100000012

200012000100000012

000020000000000000

111000100010110000

111000100010110000

111000100010110000

111000100010110000

111000100010110000

392040415240405021562827393857854432

102210123032020110

001000010030010110

001000010030010110

001000010030010110

001000010030010110

101210113002010000

000100000000010000

000100000000010000

000100000000010000

101000110000000000

101000110000000000

101000110000000000

000110003002000000

100104000002010001

100102000000000000

100102000000000000

100102000000000000

100102000000000000

000002000002010001

000002000002010001

000002000002010001

000002000002010001

0000000000000014000
16131232192814219352010292945253218

011000000002016020

011000000002016020

011000000002016020

012100200000501020

012100200000501020

010000000000301020

002100200000200000

330011100112022021

330011100112022021

330011100112022021

330011100112022021

000100110300001000

000100110300001000

000100110300001000

000100110300001000

000000000000001001

000000000000001001

000000000000001001

000000000000001001

000010000000030000
117723184101092712562116132315

200000130021140130

200000130021140130

200000130021140130

1451170213331124563

1451170213331124563

1451170213331124563

8321210476624734121271412

000700000000000000

001000201000001100

73151045652472412106145

100000000001001007

100000000001001007

002000000011003020

002000000011003020

000000000000003020

000000000000003020

002000000011000000

002000000011000000

100000000200010100

100000000200010100

100000000200010100

100000000200010100

1107023010026018411111

1107023010026018411111

1107023010026018411111

000300000140031000

1103030500201100410

0001020050000000400

000000000100710301

021022252000312402

021022252000312402

000000000000000200
021022252000312402

021022132000302202

021022132000302202

000000120000010000

000000120000010000

21524630623227215136510551111

21524630623227215136510551111

001020000000000600
2152463062322721510651055811

11132343100002001522

1112234390002001322

001000010000000200

010000023000000000

631216183217213474236

631216183217213474236

200010011111001100

20831811261326311133

20831811261326311133

000000000003000030

000000000003000030

001000000000100000

001000000000100000

001000000000100000

001000000000100000

001000000000100000

22993115613907928380227832638682233850128299176823518119455176572057826342702243194613064

000010000000000001

000010000000000001

000010000000000001

000010000000000001

000010000000000001

22993115613907928380227822638682233850128299176823518119455176572057826342702243194613063
2101031022222003037

2502322100000022110

000020000000000000

000020000000000000

000020000000000000

1502302000000021000

010100000000001000

010100000000001000

0102100000000000000
1402202000000020000

110002000000000000

020100000000020000

100000100000001110

000110000000001113

000110000000001113

000110000000001113

000110000000001113

011100020000002101

010100010000002000

010100010000002000

001000010000000101

001000010000000101
000000000000000101

001000010000000000

154251610701454591692668264224137817642342770405
22940115093903028314227332635581343845428190176063515119419176292052126281701393188813024

000000200000000000

000000200000000000

000000200000000000

601215112213311512

601215112213311512

601215112213311512

000030120330001002

000030120330001002

07116661542010191915271982

07116661542010191915271982
0511532113621213211751

000020002000010021

000001011010000100

010113402142120110

01000000972412230000

311200000100120100

110000000000110000

201200000100010100

201200000100010100

000010200000000000

000010200000000000

000010200000000000

20013993920032005220237263

000002000000000000

20013793920032005220237263

00010273919022005210127240

200035210100101123

3979842537267245552209069281028341422385611971357615967159341775810250135982196210033
31212452213125582412224262533382

00001039000000000000

00001039000000000000

3251583411608222092052861945201935318259102622761128491155214590697410700183204956
397684133725524510220676915626341372379811947357515945159101773210225135652192410031

7252579256472301153972110614784553916858143096435831423251286536045075

221835303368412827232323191220242318
024163315051210324

122320332222201220

122320332222201220

000010000000100100

000010000000100100

20132820205830191818161711715161412

20132820205830191818161711715161412

111647552303344252

111647552303344252

2664511986571653577245773773168523568149

2664511986571653577245773773168523568149
000000000000000020

25742430415728772354169101416146

937686538701073364684231503

0801412160110100002115
186422576167926353541881966922874132507829206198915652638158805621883192397

1859312921639149727717783136525741004698280891241543260615839561545369765
186402566167726213511881466732854129507429205197615652632158705619483042385

107020100000000191

60100350036301021600

117823301188252110212111

000000000600000200

294922711217299534252324363111218521925272029251618

2020122000000591804

2020122000000591804

000000000000010102

000000000000010102

000000100100000010

010010020211000031

010001002002001300

010001002002001300

512941149986171031841113

123131212030026102

000010000010014101

123121212020012001

3653826646111011011010

3653826646111011011010

000000000020000100

000000000020000100

141001122010002111

141001122010002111

4945484046267843107742834285553825328

4544453841267539105732131285553765225

000011001000000020

000011001000000020

000101002000000000

000101002000000000

56411721298926194637
55410621298824153636

000010000000030000

010100000102011001

022010000000000000

403639263122633091641925274649704718

403639263122633091641925274649704718

000010003000000000

000010003000000000

413250342173000613

303240130042000412

303240130042000412

110010212131000201
110010111131000201

000000101000000000

000000000000020000

000000000000020000

000000000000020000

000000000000020000

000000000000020000

000000000000020000

072344102131121612

071334102131121612

071334102131121612

071334102131121612

010000100001010001
010201101101010001

000201001100000000

061133001030111611

001010000000000000

001010000000000000

001010000000000000

001010000000000000

001010000000000000

000000000000000002
337145330612071343

100000110100030100

100000110100030100

100000110100030100

100000110100030100

100000110100030100

221122010512020111

201121000401010111

201121000401010111

201121000401010111

201121000401010111

020001010111010000

020001010111010000

020001010111010000

020001010111010000

001000200000000030

001000200000000030

001000200000000030

001000200000000030

001000200000000030

000000010000001000

000020000000000000

000020000000000000

000020000000000000

000020000000000000

000020000000000000

011000000000000000

011000000000000000

011000000000000000

011000000000000000

011000000000000000

004003000000020100

004003000000020100

004003000000020100

704821352642002513

704821352642002513

704821352642002513

703821342642002503

001000200110002402

001000200110002402

702821142532000101
702511042312000000

000310100220000101

001000010000000010

001000010000000010

001000010000000010

1151212827210050322

1151212827210050322

1151212827210050322

1151212827210050322

000011000000000010

000011000000000010

1151111827210050312

600000000300000100
