# Supplementary material for: Effects of dietary tryptophan supplementation on rectal temperature, humoral immunity, and cecal microflora composition of heat-stressed broilers
Source: Front Vet Sci. 2023 Sep 28;10:1247260. doi: 10.3389/fvets.2023.1247260 (PMC10572358; doi:10.3389/fvets.2023.1247260)
Supplement: Supplementary file 3 [file Data_Sheet_1.ZIP › Guanhong Li-Data report.html]

16S rDNA 扩增子测序生物信息学分析报告


<body></body>
